# Gemeinschaftsdiagnose: Ohne russisches Gas droht eine scharfe Rezession in Deutschland

**DOI:** 10.1007/s10273-022-3187-3

**Published:** 2022-05-20

**Authors:** Martin Gornig, Oliver Holtemöller, Stefan Kooths, Torsten Schmidt, Timo Wollmershäuser

**Affiliations:** 1 Abtl. Industriepolitik, Deutsches Institut für Wirtschaftsforschung, 10108 Berlin, Deutschland; 2 Abteilung Makroökonomik, Institut für Wirtschaftsforschung Halle, Kleine Märkerstraße 8, 06108 Halle (Saale), Deutschland; 3 Leiter Prognosezentrum, Institut für Weltwirtschaft, Kiellinie 66, 24105 Kiel, Deutschland; 4 RWI - Leibniz-Institut für Wirtschaftsforschung, Postfach 10 30 54, 45030 Essen, Deutschland; 5 Befragungen, ifo Zentrum für Konjunkturforschung, Poschingerstraße 5, 81679 München, Deutschland

**Keywords:** E27, E32, E37

## Abstract

Die deutsche Wirtschaft steuert durch schwieriges Fahrwasser. Die Auftriebskräfte durch den Wegfall der Pandemiebeschränkungen, die Nachwehen der Coronakrise und die Schockwellen durch den Krieg in der Ukraine sorgen für gegenläufige konjunkturelle Strömungen. Allen Einflüssen gemeinsam ist ihre preistreibende Wirkung.

## Abstract (en)

The German economy is steering through difficult waters. Tail winds from fading pandemic restrictions, supply-side bottlenecks in the aftermath of the coronavirus crisis, and shock waves caused by the war in Ukraine are dragging the economy in opposing directions. The common factor is the price-driving effect. Abruptly stopping gas deliveries from Russia to the European Union would drive the German economy into a deep recession. In this case, the accumulated loss of overall economic output would amount to 220 billion euro by the end of 2023.

Nach dem Überfall Russlands auf die Ukraine hat der Westen – darunter alle EU-Mitgliedsländer – umfangreiche Sanktionen gegen die Russische Föderation in Kraft gesetzt, von dem allerdings der Rohstoffhandel bislang weitgehend ausgenommen ist, insbesondere Erdgas. Im Zuge der Zuspitzung des Konflikts steht zunehmend die Möglichkeit im Raum, dass die Lieferungen für Erdöl und Erdgas von einer der beiden Seiten gestoppt werden. In ihrem Frühjahrsgutachten[1] haben die Institute den Fall eines sofortigen Lieferstopps in einem Alternativszenario abgebildet. Anders als im Basisszenario, bei dem die Erholungskräfte im Zuge des Abklingens der Pandemie mehr und mehr die Oberhand gewinnen, würde die deutsche Wirtschaft 2023 in eine scharfe Rezession geraten.

## Basisszenario

Die mit der abflauenden Pandemie einhergehende wirtschaftliche Erholung wird insbesondere durch die kontaktintensiven Dienstleistungsbereiche getragen. Dort können mit dem Wegfall der meisten Infektionsschutzmaßnahmen die Kapazitäten wieder stärker genutzt werden. Zugleich dürfte die Nachfrage rege sein, auch weil viele Dienstleistungen über längere Zeit entbehrt werden mussten. Zudem haben die privaten Haushalte während der Pandemie Überschussersparnisse von rund 200 Mrd. Euro gebildet, weil sie ihre gewohnten Verbrauchsmuster pandemiebedingt nicht beibehalten konnten. Das hohe Ausmaß an weltweit aufgestauter Kaufkraft wirkt belebend auf die Konjunktur und dürfte einen Gutteil des derzeitigen Inflationsdrucks erklären. Zu diesem Inflationsdruck hat die Finanzpolitik beigetragen, indem sie während der Pandemie die privaten Einkommen in erheblichem Umfang stabilisiert hat. Flankierend wirkte eine ultralockere Geldpolitik, die auch jetzt kaum gegensteuert – aktuell senkt die in die Höhe geschnellte Inflation die ohnehin negativen Realzinsen nochmals deutlich. Im Zuge der Pandemie wurden die internationalen Lieferketten erheblich gestresst. In der Folge war auch die deutsche Industrie mit Materialengpässen historischen Ausmaßes konfrontiert, während sich die Nachfrage ausweislich des regen Auftragseingangs bereits kräftig erholt hatte. Insgesamt dürfte sich der Auftragsüberhang im Verarbeitenden Gewerbe mittlerweile auf 100 Mrd. Euro belaufen. Zwar ist die Industrieproduktion seit fünf Monaten wieder aufwärtsgerichtet, gleichwohl belasten Lieferengpässe

1 Vgl. Projektgruppe Gemeinschaftsdiagnose (2022a).

**Prof. Dr. Martin Gornig** ist Forschungsdirektor Industriepolitik und stellvertretender Leiter der Abteilung Unternehmen und Märkte am Deutschen Institut für Wirtschaftsforschung (DIW).

**Prof. Dr. Oliver Holtemöller** ist stellvertretender Präsident und leitet die Abteilung Makroökonomik am Leibniz-Institut für Wirtschaftsforschung Halle (IWH).

**Prof. Dr. Stefan Kooths** ist Direktor des Forschungszentrums Konjunktur und Wachstum am Kiel Institut für Weltwirtschaft (IfW).

**Prof. Dr. Torsten Schmidt** leitet den Kompetenzbereich Wachstum, Konjunktur, Öffentliche Finanzen am RWI – Leibniz-Institut für Wirtschaftsforschung.

**Prof. Dr. Timo Wollmershäuser** ist stellvertretender Leiter des ifo Zentrums für Makroökonomik und Befragungen.

weiterhin die industrielle Aktivität. Im März dürfte die Produktion sogar infolge ausbleibender Zulieferungen aus der Ukraine deutlich nachgegeben haben. Hohe Nachfrage bei gehemmtem Angebot macht sich in stärkerem Preisdruck geltend. In dem Maße, wie Lieferengpässe nach und nach überwunden werden, lassen die Nachholeffekte auch in der Industrie für sich genommen einen selbsttragenden Aufschwung erwarten.

Die ab dem Frühjahr mit dem Überwinden der Pandemie und ihrer Folgen angelegte kräftige Erholung wird durch den Kriegsausbruch in der Ukraine zunächst gebremst. Der Krieg und die politischen Reaktionen wirken angebots- und nachfrageseitig über verschiedene Kanäle als negativer Schock auf die wirtschaftliche Aktivität. Spürbar, aber gesamtwirtschaftlich von untergeordneter Bedeutung, ist der Wegfall der Exportmärkte, der insbesondere mit den westlichen Sanktionen gegenüber Russland verbunden ist. Bedeutender ist die massiv gestiegene Unsicherheit über die Rohstoffversorgung, insbesondere – aber nicht nur – bei wichtigen Energierohstoffen, die den bereits vor dem russischen Überfall auf die Ukraine in Gang gekommenen Preisauftrieb weiter angefacht hat. Damit fließt über die höhere Energieimportrechnung entsprechend mehr Kaufkraft ins Ausland ab und schwächt hierzulande die Nachfrage. Zugleich kommt es durch die kriegsbedingten Störungen zu neuen Lieferengpässen, die kurzfristig nicht zuletzt die Automobilindustrie treffen. Im zurückliegenden Winterhalbjahr haben vor allem die Maßnahmen zum Infektionsschutz die Wirtschaftsleistung gedämpft. Unter der Voraussetzung, dass das Kriegsgeschehen in der Ukraine die ökonomische Aktivität nicht noch stärker belastet, dürften die konjunkturellen Auftriebskräfte ab dem Frühjahr die Oberhand gewinnen (vgl. Abbildung 1). Nach einem schwachen Jahresauftakt dürfte das Bruttoinlandsprodukt (BIP) im zweiten Quartal zwar deutlich zulegen, ohne die Belastung durch den Krieg in der Ukraine würde das Plus aber kräftiger ausfallen. Insgesamt verzögert sich damit der Erholungsprozess abermals. Das Vorkrisenniveau der Wirtschaftsleistung wird erst im dritten Quartal 2022 erreicht werden und damit ein halbes Jahr später, als von den Instituten in ihrem Herbstgutachten 2021 erwartet worden war. Alles in allem erwarten die Institute einen Anstieg des BIP um 2,7 % für dieses Jahr und um 3,1 % für nächstes Jahr. Gleichwohl ist die Expansionsdynamik im laufenden Jahr stärker als im kommenden Jahr, in dem die deutsche Wirtschaft in eine leichte Überauslastung driftet. Maßgeblich dafür sind der hohe Auftragsüberhang in der Industrie sowie nachholende Konsumaktivität.

Die Verbraucherpreise ziehen 2022 mit einer Rate von 6,1 % so kräftig an wie seit 40 Jahren nicht mehr (vgl. Tabelle 1). Auch im kommenden Jahr bleibt die Rate mit

Abbildung 1

**Szenarienvergleich – Reales BIP in Deutschland**

Verkettete Volumenangaben in Mrd. Euro; saison- und kalenderbereinigt

Quellen: Statistisches Bundesamt; Berechnungen der Institute; ab dem ersten Quartal 2022: Prognose der Institute.

2,8 % deutlich über dem Durchschnitt seit der Wiedervereinigung. Zwar verschärft der Krieg in der Ukraine den Preisauftrieb, der Prozess einer sich beschleunigenden Inflation hat jedoch schon vor einem Jahr eingesetzt. Dabei kommen die starken Rohstoffpreisanstiege erst nach und nach auf der Verbraucherstufe an. Allerdings treiben nicht nur höhere Energiepreise die Teuerung. So nimmt der heimische Preisdruck – gemessen am Deflator des BIP – in beiden Prognosejahren mit über 3 % deutlich zu, und auch die Kernrate der Inflation dürfte im kommenden Jahr noch bei 3,1 % liegen. Insgesamt hat sich ein breit angelegter Preisdruck aufgebaut, der auch dann noch nachwirkt, wenn annahmegemäß die Rohstoffpreise wieder etwas nachgeben und die Lieferengpässe in der zweiten Jahreshälfte sukzessive nachlassen.

## Alternativszenario: Lieferstopp für russisches Gas

### Weltwirtschaftliches Umfeld

Bei einem Stopp der europäischen Gas- und Öllieferungen aus Russland verschlechtern sich insgesamt die Rahmenbedingungen für die Weltwirtschaft. Zum einen ziehen die Weltmarktpreise für Öl und Gas nochmals deutlich an, was weltweit die Konjunktur bremst. Zum anderen ist in der EU zeitweise nicht mehr ausreichend Gas vorhanden, um die Produktion in dem Umfang umzusetzen, wie sie im Basisszenario erwartet wird. Die dadurch verringerte ökonomische Aktivität in Europa wird angesichts der Größe des Wirtschaftsraums insbesondere über die Handelsverflechtung auch in der übrigen Welt spürbar.

Bei einem Lieferstopp von Rohöl müssen weltweit schätzungsweise täglich 4 Mio. Barrel Öl und 1,5 Mio. Barrel Ölprodukte, die bislang aus Russland kamen, aus ande-

Tabelle 1
**Eckdaten der Prognose für Deutschland**

| | Basisszenario | | | Alternativszenario | |
|---|---|---|---|---|---|
| | 2021 | 2022 | 2023 | 2022 | 2023 |
| Reales BIP (Veränderung gegenüber dem Vorjahr in %) | 2,9 | 2,7 | 3,1 | 1,9 | -2,2 |
| Erwerbstätige im Inland in 1.000 Personen | 44.920 | 45.454 | 45.733 | 45.370 | 45.185 |
| Arbeitslose in 1.000 Personen | 2.613 | 2.293 | 2.293 | 2.369 | 2.787 |
| Arbeitslosenquote der Bundesagentur für Arbeit[1] in % | 5,7 | 5,0 | 5,0 | 5,2 | 6,0 |
| Verbraucherpreise[2] (Veränderung gegenüber dem Vorjahr in %) | 3,1 | 6,1 | 2,8 | 7,3 | 5,0 |
| Lohnstückkosten[3] (Veränderung gegenüber dem Vorjahr in %) | 0,5 | 2,2 | 2,1 | 2,0 | 3,2 |
| Finanzierungssaldo des Staats[4] | | | | | |
| in Mrd. Euro | -132,5 | -52,2 | -27,9 | -76,2 | -157,5 |
| in % des nominalen BIP | -3,7 | -1,4 | -0,7 | -2,0 | -4,1 |
| Leistungsbilanzsaldo | | | | | |
| in Mrd. Euro | 265,3 | 214,2 | 245,8 | 187,6 | 117,0 |
| in % des nominalen BIP | 7,4 | 5,6 | 6,1 | 5,0 | 3,1 |

[1] Arbeitslose in % der zivilen Erwerbspersonen (Definition gemäß Bundesagentur für Arbeit). [2] Verbraucherpreisindex (2015 = 100). [3] Im Inland entstandene Arbeitnehmerentgelte je Arbeitnehmerstunde bezogen auf das reale BIP je Erwerbstätigenstunde. [4] In der Abgrenzung der Volkswirtschaftlichen Gesamtrechnungen (ESVG 2010).

Quellen: Statistisches Bundesamt; Bundesagentur für Arbeit; Deutsche Bundesbank; 2022 und 2023: Prognose der Institute.

ren Quellen gedeckt werden (McWilliams et al., 2022). Die Institute rechnen in diesem Szenario damit, dass der Preis für Öl der Sorte Brent zunächst kräftig steigt, auf 135 US-$ je Barrel im Durchschnitt des zweiten Quartals 2022, sich im weiteren Verlauf dann aber sukzessive verringert und im zweiten Halbjahr des kommenden Jahres wieder auf den Preispfad des Basisszenarios einschwenkt (vgl. Abbildung 2). Grund dafür ist neben der angeregten Mehrproduktion auch die Eintrübung der weltwirtschaftlichen Aktivität.

Im Fall von Erdgas ist mit einem stärkeren und nachhaltigeren Anstieg der Weltmarktpreise zu rechnen. Neben dem Rückgriff auf die sehr begrenzten zusätzlichen Kapazitäten aus anderen leitungsverbundenen Lieferländern wie Norwegen oder Algerien wird die europäische Nachfrage nach Flüssiggas drastisch zunehmen. Dieses wird weltweit gehandelt und steigende Preise treffen damit auch die großen Importländer in Asien, aber auch Länder in Lateinamerika. Zusätzliche Flüssiggaskapazitäten werden nur langsam an den Markt kommen, und die Umleitung des russischen Pipeline-Gases zu anderen Nachfragern ist kurzfristig kaum möglich. Für die Prognose rechnen die Institute mit einem europäischen Erdgaspreis, der ausgehend von 200 Euro je MWh im zweiten Quartal nur allmählich sinkt und auch am Ende des Prognosezeitraums noch doppelt so hoch ist wie im Basisszenario.

Abbildung 2
**Energiepreisannahmen**

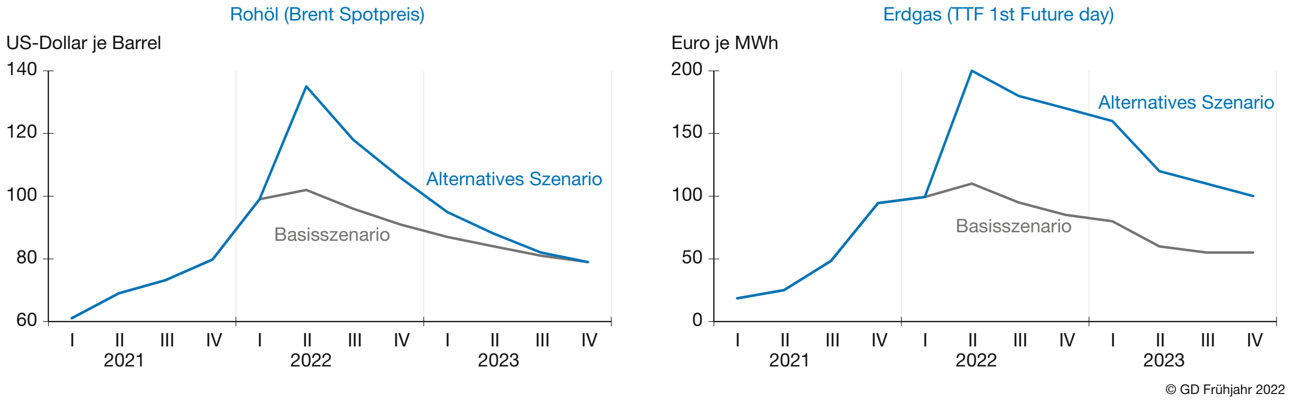

Quellen: Refinitiv; Prognose der Institute.

Die höheren Energiepreise bremsen weltweit die Konjunktur. In Europa kommt hinzu, dass in einer Reihe von Ländern russisches Gas als Rohstoff so wichtig ist, dass es kurzfristig nicht vollständig ersetzt werden kann und – wie in Deutschland – mit Rationierungen zu rechnen ist, welche die Industrieproduktion zeitweise deutlich sinken lassen werden.

Alles in allem rechnen die Institute damit, dass das BIP in der EU 2022 mit einer Rate von 3 % um 0,3 Prozentpunkte schwächer steigt als im Basisszenario (vgl. Tabelle 1). Da sich die negativen Wirkungen des Importstopps erst nach und nach entfalten und die Effekte der Rationierung sich im Winter konzentrieren, ist der Abschlag gegenüber dem Basisszenario im kommenden Jahr mit 2,5 Prozentpunkten erheblich größer und der verbleibende Produktionszuwachs mit 0,2 % nur noch gering. Die Weltproduktion insgesamt fällt 2022 um 0,3 % niedriger aus als im Basisszenario. 2023 reduziert sich die Expansionsrate der Weltwirtschaft um 1 Prozentpunkt, gewichtet mit deutschen Exportanteilen sogar um 1,8 Prozentpunkte. Bei alledem wird die Inflation nochmals spürbar höher sein als im Basisszenario.

## Auswirkungen auf die deutsche Wirtschaft

Wie in anderen Ländern wirkt die Unterbrechung der Gasversorgung aus Russland auch in Deutschland als negativer Angebotsschock. Die Institute leiten die Stärke des Schocks und seine Folgen in fünf Schritten ab:[2]

- zeitliches Profil der Gasverfügbarkeit
- unmittelbare Produktionsausfälle im Verarbeitenden Gewerbe
- Verstärkungs- und Ausstrahleffekte auf andere Wirtschaftsbereiche
- Kaufkraftentzug infolge höherer Energiepreise
- gesamtwirtschaftliche Kreislaufanalyse

Das zeitliche Profil der Gasverfügbarkeit ergibt sich aus dem Zusammenspiel von Produktion, Verbrauch und den Speicherfüllständen. Dabei ist auf der Basis unterschiedlicher angebots- und nachfrageseitiger Substitutionspotenziale (Holz et al., 2022; Bundesverband der Energie- und Wasserwirtschaft e. V., 2022) eine Vielzahl plausibler Kombinationen vorstellbar. Anstatt eine einzige Konfiguration auszuwählen, haben die Institute im Rahmen einer stochastischen Simulation einen Medianverlauf für die Gasverfügbarkeit bestimmt, der somit auf halbem Weg zwischen ungünstigster und günstigster Konstellation liegt. Die privaten Haushalte werden in allen betrachteten Konstellationen dem „Notfallplan Gas" folgend nicht rationiert. Die Institute unterstellen allerdings, dass die Gasspeicher nicht wie bislang vorgesehen gefüllt werden, sondern dass versucht wird, Produktionsausfälle in der Industrie zu reduzieren. Zur Schätzung der Unsicherheit bezüglich der Gasproduktion und des Verbrauchs wurden 1.000 Simulationsläufe für unterschiedliche Produktions- und Verbrauchsmengen durchgeführt. Unter günstigen Bedingungen kann eine Mangellage vermieden werden; mit großer Wahrscheinlichkeit reichen die in den Gasspeichern vorgehaltenen Mengen jedoch nicht für eine unterbrechungsfreie Versorgung der Industrie aus (vgl. Abbildung 3). So werden ab dem Jahreswechsel 2022/2023, wenn in der Heizperiode die Vorräte aufgezehrt sind, für einen Zeitraum von vier Monaten (Januar bis April 2023) nicht mehr alle industriellen Verbraucher wie gewohnt beliefert werden können. Den Simulationen zufolge würden im Winter 2023/2024 unter unveränderten Parametern abermals Engpässe auftreten. Die Institute nehmen an, dass die längere Vorbereitungszeit Substitutionsmöglichkeiten erschließt, die die Versorgungsengpässe im Vergleich zum Winter 2022/2023 deutlich mildern dürften.

Im Verarbeitenden Gewerbe wird der größte Gasanteil für Prozesswärme benötigt oder fließt direkt in die Verarbeitung ein, sodass dort der Verzicht auf Erdgas zu Produktionsausfällen führt. Die Gasmengen werden in dem hier simulierten Rationierungsregime den einzelnen Industriezweigen proportional gekürzt. Dies führt

Abbildung 3

**Umfang der Gasrationierung im Verarbeitenden Gewerbe bei Importstopp russischer Brennstoffe**

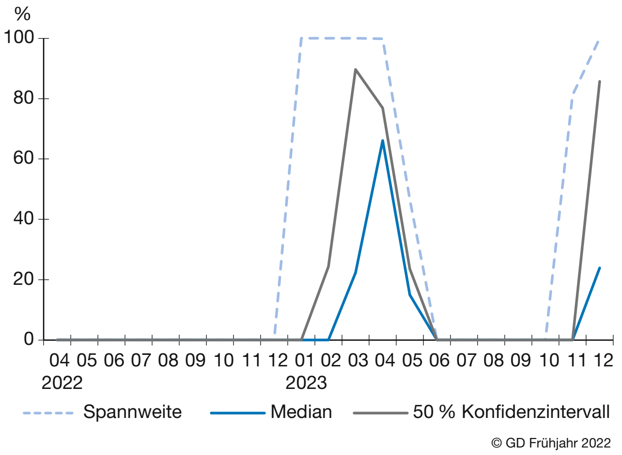

Quellen: AG Energiebilanzen e. V.; OECD; Simulation der Institute.

2 Für eine detaillierte Beschreibung der Vorgehensweise vgl. Projektgruppe Gemeinschaftsdiagnose (2022b).

zu Produktions- und Wertschöpfungsverlusten, die der prozentualen Reduktion der Gasversorgung entsprechen, gewichtet mit den branchenspezifischen Erdgasanteilen an ihrem jeweiligen Endenergieverbrauch.[3] Diese Produktionsausfälle strahlen als Zweitrundeneffekt über die Vorleistungsverflechtung auf die nachgelagerte Produktionsstufe aus, die anhand einer Input-Output-Tabelle bestimmt werden. Dabei wird angenommen, dass ein Wirtschaftsbereich nur dann die Produktion zusätzlich drosseln muss, wenn die Einschränkung durch fehlende Vorleistungen die aufgrund der Gasknappheit bestehende Einschränkung aus dem Erstrundeneffekt übersteigt.[4] Die Institute gehen davon aus, dass sich die resultierenden Zweitrundeneffekte kurzfristig nicht durch ausländische Lieferungen kompensieren lassen. Insgesamt ergibt sich für den Verlauf der Gasrationierung ein Rückgang der Wertschöpfung im Verarbeitenden Gewerbe von rund 13,5 % im zweiten Quartal 2023 gegenüber dem vierten Quartal 2022 (vgl. Abbildung 4, Kurve IO-Impuls). Etwa zur Jahresmitte 2023 ist in diesem Szenario wieder Erdgas in ausreichendem Umfang verfügbar, sodass die Produktion wieder hochgefahren wird.

Abbildung 4
**Bruttowertschöpfung im Verarbeitenden Gewerbe**
Verkettete Volumenangaben in Mrd. Euro; saison- und kalenderbereinigt

Quellen: Statistisches Bundesamt; Berechnungen der Institute; ab dem ersten Quartal 2022: Prognose der Institute.

Solche Produktionseinbrüche wirken typischerweise im Verarbeitenden Gewerbe nach, weil dadurch Lieferketten erheblich gestresst werden. Zudem strahlen sie auch auf andere Wirtschaftsbereiche aus. Diese Effekte, wie auch die anschließende Erholung, quantifizieren die Institute anhand einer Mittelung verschiedener Modellergebnisse. Verwendet wurden unter anderem ein ARMA-Modell für die Wertschöpfung im Verarbeitenden Gewerbe und ein Zeitreihenmodell für die Effekte eines Ölangebotsschocks, die beide ähnliche Ergebnisse zeigen. Die Unterauslastung im Verarbeitenden Gewerbe wird sich demnach über mehrere Quartale hinziehen (vgl. Abbildung 4, Kurve Alternativszenario).

Nach einem Dämpfer im Auftaktquartal 2023, bricht das BIP im zweiten Quartal um fast 5 % ein (vgl. Abbildung 1). Eine Erholung im Schlussquartal fängt dies zwar teilweise auf, alles in allem sinkt das BIP 2023 aber um fast 2 % – gegenüber einem gut 3 %igen Anstieg im Basisszenario. Der kumulierte Verlust an gesamtwirtschaftlicher Produktion dürfte sich bereits in beiden Jahren auf rund 220 Mrd. Euro belaufen, was 6,5 % der jährlichen Wirtschaftsleistung entspricht.

Das hier gezeigte Produktionsmuster hängt entscheidend vom Rationierungsregime ab. Die Institute gehen davon aus, dass zeitgleich mit dem Lieferstopp die Notfallstufe des „Notfallplans Gas“ in Kraft tritt. Annahmegemäß werden die Mindestfüllstände außer Kraft gesetzt, ein Mehrbezug von Gas gegenüber dem Vorjahresniveau dürfte jedoch unterbunden werden. Damit ist ein Vorziehen der Produktion nicht möglich. Allerdings fehlt bislang auch ein Mechanismus, der es einem industriellen Verbraucher ermöglichen würde, durch eine vorgezogene Reduktion des Gasverbrauchs Reserven für die Engpassphase zu bilden. In der Folge wird die Produktion der gasverbrauchenden Industrie im Vorfeld des Engpasses noch nicht angepasst.

Die Verbraucherpreise werden weitaus stärker steigen als im Basisszenario (vgl. Abbildung 5). Maßgeblich sind die spürbar höheren Rohstoffpreise. Vor allem die Preise für Gas, das zuletzt 2,5 % des Warenkorbs der Verbrauchenden ausmachte, steigen kräftig. Höhere Marktpreise für Gas machen sich in der Verbraucherpreisinflation aufgrund bestehender Lieferverträge für gewöhnlich erst nach und nach bemerkbar. Daher werden die höheren Gaspreise in weiten Teilen des Prognosezeitraums die Verbraucherpreisinflation anfachen. Neben den damit verbundenen unmittelbar höheren Energiepreisen werden die Verbraucherpreise zusätzlich durch die angesichts der höheren Rohstoffpreise deutlich anziehenden Erzeu-

3 Verknappt sich beispielsweise die Gasmenge in einem Industriezweig, der ein Zehntel seines Energieverbrauchs mit Erdgas deckt, um die Hälfte, muss dessen Wertschöpfung um 5 % gedrosselt werden.

4 Diese Annahme bedeutet, dass die von beiden Effekten betroffenen Unternehmen übereinstimmen: Führen beispielsweise die Gasknappheit zu einem Wertschöpfungsausfall von 10 % und die fehlenden Vorleistungen zu einem Ausfall von 5 % (oder umgekehrt), wird davon ausgegangen, dass insgesamt 10 % ausfallen. Im anderen Extrem betreffen die Gas- und die Vorleistungsknappheit unterschiedliche Unternehmen; dann würden sich beide Effekte voll (additiv) entfalten.

Abbildung 5
**Szenarienvergleich – Verbraucherpreise**
Veränderung gegenüber dem Vorjahr in %

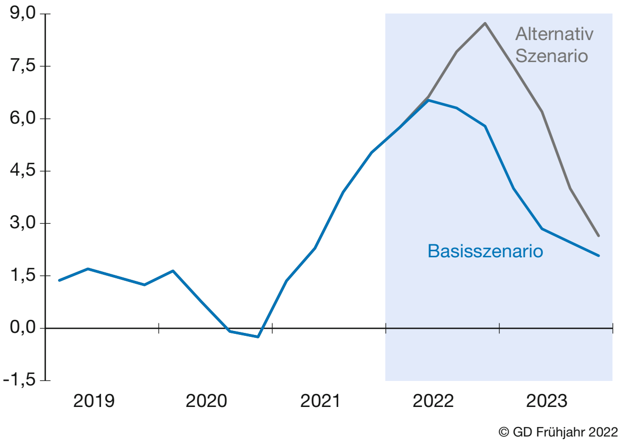

Quellen: Statistisches Bundesamt; Berechnungen der Institute; ab dem ersten Quartal 2022: Prognose der Institute.

gerpreise steigen. Insgesamt dürfte die Inflation 2022 bei 7,3 % liegen (Basisszenario: 6,1 %) und 2023 mit 5 % (2,8 %) weiter ausgesprochen hoch bleiben.

Der Außenhandel wird durch ein Gasembargo spürbar belastet. Bereits im laufenden Jahr dämpft die schwächere wirtschaftliche Entwicklung in den deutschen Absatzmärkten die Exporte gegenüber dem Basisszenario. 2023 brechen die Exporte vorübergehend ein, da die Produktion durch den Mangel an Erdgas massiv gestört wird. Die Erholung der Produktion, die sich mit dem Nachlassen der Engpässe gegen Ende des Jahres 2023 einstellt, übersetzt sich auch in die Exporte. Insgesamt rechnen die Institute damit, dass die Exporte im Jahr 2022 um 4,2 % steigen und 2023 um 3,5 % zurückgehen. Das sind 0,7 (2022) bzw. 8,1 Prozentpunkte (2023) weniger als im Basisszenario. Auch die Importe leiden im laufenden Jahr gegenüber dem Basisszenario. So fallen die Einfuhren von Erdgas aus Russland im zweiten Quartal weg und werden zunächst nicht ersetzt. Zugleich wirken sich die noch deutlich höheren Energiepreise im gesamten Prognosezeitraum negativ auf die Binnenwirtschaft aus. Hinzu kommt der Effekt der Produktionsausfälle im ersten Halbjahr 2023. Alles in allem ergibt sich ein Anstieg der Importe um 4,6 % im Jahr 2022 (-0,9 Prozentpunkte) und ein Rückgang um 0,3 % im Jahr 2023 (-5,5 Prozentpunkte). Die Terms of Trade verschlechtern sich im Prognosezeitraum aufgrund der höheren Energiepreise deutlich stärker als im Basisszenario.

Der starke Produktionsrückgang im Verarbeitenden Gewerbe dürfte auch dazu führen, dass die Ausrüstungsinvestitionen im zweiten Quartal 2023 um 7 % gegenüber dem Vorquartal einbrechen. In Erwartung der Produktionsdrosselung werden sie aber bereits in diesem Jahr mit 0,7 % etwas schwächer ausgeweitet (-0,3 Prozentpunkte). Im folgenden Jahr dürften die Ausrüstungen nur um 3,1 % (-10,3 Prozentpunkte) zulegen. Auch die Investitionen in Bauten dürften durch ein Gasembargo bereits im laufenden Jahr etwas schwächer zulegen. Fehlende Vorleistungen betreffen alle Bausparten. Zusätzlich wird der Wirtschaftsbau durch die Schwäche im Verarbeitenden Gewerbe belastet. Im Folgejahr dürften die Bauinvestitionen leicht rückläufig sein (-1% bzw. -3 Prozentpunkte).

Der private Verbrauch wird in beiden Jahren eingeschränkt, weniger aufgrund physischer Knappheiten, sondern infolge von Kaufkraftverlusten. Die verfügbaren Realeinkommen fallen gegenüber dem Basisszenario 2022 um 1,4 % niedriger aus, im nächsten Jahr um 7,0 %. In diesem Jahr belastet vor allem die höhere Inflation (+1,2 Prozentpunkte), die bei kaum geringerem Nominaleinkommen bereits deutlich auf den realen Konsum durchschlägt (-1,4 %). Die im kommenden Jahr deutlich geringeren verfügbaren Einkommen (-4,0 %) – die geringere Lohnsumme (-4,9 %) wird nur teilweise durch höhere Sozialleistungen aufgefangen, zudem brechen Einkommen aus selbstständiger Arbeit und Vermögen weg (-11,5 %) – und eine höhere Teuerung (+2,2 Prozentpunkte) schlagen sich in einem spürbar geringeren Konsum (-6,4 %) nieder.

Am Arbeitsmarkt hinterlässt der Lieferstopp ebenfalls deutliche Spuren. Die im Vergleich zum Basisszenario gedämpfte wirtschaftliche Aktivität 2022 führt zu einer verringerten Arbeitsnachfrage der Unternehmen. Da es 2022 zu einer graduellen Verschlechterung der wirtschaftlichen Lage kommt sowie im Zuge der höheren Unsicherheit und in Erwartung des Produktionseinbruchs im Jahr 2023, dürfte sich die verringerte Arbeitsnachfrage weniger in der Arbeitszeit je Erwerbstätigen, sondern eher über weniger Neueinstellungen niederschlagen. Da die Unternehmen davon ausgehen, dass der Produktionseinbruch 2023 nur vorübergehend ist, wird der Arbeitsausfall überwiegend über eine reduzierte Arbeitszeit je Beschäftigten – und eine damit verbundene massive Ausweitung der Kurzarbeit – bewerkstelligt. Dadurch können die Unternehmen ihre Aktivität nach Ende der Versorgungsknappheit rasch wieder hochfahren. Die Institute rechnen im Durchschnitt des Jahres 2023 mit einem Kurzarbeiter-Beschäftigtenäquivalent von 725.000.[5] Die Erwerbstätigkeit fällt Ende des Jahres 2023 um 750.000 Personen niedriger aus als im Basisszenario. Die Arbeitslosenquote steigt von durchschnittlich 5,2 % (2022) auf

5 Zum Vergleich: 2020 lag das Kurzarbeiter-Beschäftigtenäquivalent bei knapp 1,2 Mio. Personen.

6,0 % (2023) – im Basisszenario liegt sie in beiden Jahren bei 5,0 %. Die Lohnentwicklung wird deutlich schwächer ausfallen als im Basisszenario. Trotz der höheren Preisdynamik fällt der Verteilungsspielraum aufgrund der Rezession deutlich kleiner aus. Darüber hinaus werden die Verdienste durch die massive Ausweitung der Kurzarbeit vorübergehend gedrückt.

Die konjunkturelle Krise belastet die öffentlichen Haushalte merklich. Verglichen mit dem Basisszenario fallen höhere monetäre Sozialleistungen in Form von Arbeitslosen- und Kurzarbeitergeld an. Die Einnahmen aus Steuern und Beiträgen fallen deutlich geringer aus. Umfangreiche Konjunkturmaßnahmen, wie finanzielle Hilfen an besonders betroffene Branchen oder Unternehmen, sind in dieser Prognose nicht berücksichtigt. Analog zur Coronakrise gehen die Institute allerdings davon aus, dass die bestehenden Sonderregeln zur Kurzarbeit in Kraft bleiben und die Sozialversicherungen im Krisenjahr 2023 Zuschüsse vom Bund erhalten, um einen deutlichen Anstieg der Beitragssätze zu vermeiden. In der Summe dürfte das Defizit im Jahr 2022 etwas schwächer sinken als im Basisszenario und 2023 in etwa auf das Niveau der Coronakrise zurückschnellen.

## Ausblick

Der durch den Lieferstopp ausgelöste Wirtschaftseinbruch wird bis zum Ende des Prognosezeitraums noch nicht wieder aufgeholt. Im Schlussquartal verbleibt gegenüber dem Basisszenario eine Lücke von rund 4 %. Auch ein abermaliger Rückschlag im Winter 2023/2024 ist nicht auszuschließen. In der mittleren Frist dürfte sich die Wirtschaftsleistung jedoch allmählich dem Pfad annähern, der auch für das Basisszenario gilt. Maßgeblich hierfür ist das Produktionspotenzial, das weniger durch die temporäre Gaskrise als vielmehr dadurch beeinträchtigt wird, dass Energie auf absehbare Zeit am Standort Deutschland merklich teurer sein wird, als es sich vor dem Angriff Russlands auf die Ukraine darstellte. Denn die Entscheidung, unabhängig von russischen Rohstofflieferungen zu werden, dürfte auch dann noch Bestand haben, wenn sich die militärische und politische Lage wieder beruhigt. Damit muss sich ein Teil der Energieversorgung und der energieintensiven Industrie neu ausrichten.

**Title:** ***Without Russian Gas, a Sharp Recession Looms in Germany***
